# Supplementary material for: PBMCs as Tool for Identification of Novel Immunotherapy Biomarkers in Lung Cancer
Source: Biomedicines. 2024 Apr 5;12(4):809. doi: 10.3390/biomedicines12040809 (PMC11048624; doi:10.3390/biomedicines12040809)
Supplement: Supplementary file 1 [file biomedicines-12-00809-s001.zip › biomedicines-2919027-supplementary.pdf]

Supplementary Materials for

PBMCs as tool for identification of novel immunotherapy biomarkers in lung cancer

Caterina De Rosa[1,†], Francesca Iommelli[2,†], Viviana De Rosa[2,*], Giuseppe Ercolano[3], Federica Sodano[3], Concetta Tuccillo[1], Luisa Amato[1], Virginia Tirino[4,5], Annalisa Ariano[1], Flora Cimmino[6], Gaetano di Guida[1], Gennaro Filosa[1], Alessandra di Liello[1], Davide Ciardiello[7], Erika Martinelli[1], Teresa Troiani[1], Stefania Napolitano[1], Giulia Martini[1], Fortunato Ciardiello[1], Federica Papaccio[8], Floriana Morgillo[1,††], Carminia Maria Della Corte[1,††,*]

[1]Department of Precision Medicine, University of Campania Luigi Vanvitelli, Italy; caterina.derosa1@unicampania.it, concetta.tuccillo@unicampania.it, luisa.amato@unicampania.it, annalisaariano98@outlook.it, gaetano.diguida@studenti.unicampania.it, gennaro.filosa@unicampania.it, alessandradiliello@gmail.com, erika.martinelli@unicampania.it, teresa.troiani@unicampania.it, stefania.napolitano@unicampania.it, giulia.martini@unicampania.it, fortunato.ciardiello@unicampania.it, floriana.morgillo@unicampania.it, carminiamaria.dellacorte@unicampania.it
[2]Institute of Biostructures and Bioimaging, National Research Council, Naples, Italy; francesca.iommelli@ibb.cnr.it, viviana.derosa@ibb.cnr.it
[3]Department of Pharmacy, School of Medicine, University of Naples Federico II, Italy; giuseppe.ercolano@unina.it, federica.sodano@unina.it
[4]Department of Experimental Medicine, University of Campania Luigi Vanvitelli, Italy; virginia.tirino@unicampania.it
[5]U.P. Diagnostica Citometrica e Mutazionale, A.O.U. Vanvitelli, Università degli Studi della Campania, Naples, Italy
[6]Hospital "Martiri Di Villa Malta", Sarno, Campania, Italy; floracimmino81@gmail.com
[7]Division of Gastrointestinal Medical Oncology and Neuroendocrine Tumors, European Institute of Oncology (IEO), IRCCS, Milan, Italy; davide.ciardiello@unicampania.it
[8]Department of Medicine, Surgery and Dentistry, "Scuola Medica Salernitana", University of Salerno, Baronissi, Italy; fpapaccio@unisa.it

*Correspondence: viviana.derosa@ibb.cnr.it; tel +390812203430 and carminiamaria.dellacorte@unicampania.it; tel.: +393929160541
† These authors contributed equally to this work as co-first authors
†† These authors contributed equally to this work as co-last authors

The PDF file includes:

Table S1. Cytokine assay characteristics

Cytokine	Vendor	Range standard curve	Limit of detection
CCL5	Invitrogen	51,2-2000 pg/ml	2 pg/ml
CXCL10	Invitrogen	7.8-500 pg/ml	2 pg/ml

Table S2. Correlation analysis in lung cancer patients (BR, R and NR) between STING/cGAS mRNA level and serum CXCL10 and CCL5

	BR				
	STING vs. CXCL10	STING vs. CCL5	cGAS vs. CXCL10	cGAS vs. CCL5	CXCL10 vs. CCL5
**r value**	0.2855	-0.1014	0.02951	-0.4408	-0.2404
**95% CI**	-0.6847 to 0.8907	-0.8436 to 0.7738	-0.8012 to 0.8214	-0.9224 to 0.5773	-0.8802 to 0.7096
**p value**	0.5834	0.8484	0.9557	0.3816	0.6463

	R				
	STING vs. CXCL10	STING vs. CCL5	cGAS vs. CXCL10	cGAS vs. CCL5	CXCL10 vs. CCL5
**r value**	-0.1615	0.1426	-0.2599	0.2024	0.4792
**95% CI**	-0.7777 to 0.6129	-0.6249 to 0.7699	-0.8152 to 0.5445	-0.5858 to 0.7939	-0.3404 to 0.8850
**p value**	0.7023	0.7363	0.5343	0.6307	0.2295

	NR				
	STING vs. CXCL10	STING vs. CCL5	cGAS vs. CXCL10	cGAS vs. CCL5	CXCL10 vs. CCL5
**r value**	0.1732	0.4973	0.7252	0.6996	0.8396
**95% CI**	-0.6669 to 0.8194	-0.5269 to 0.9325	-0.06129 to 0.9561	-0.2590 to 0.9639	0.08816 to 0.9820
**p value**	0.7104	0.3155	0.0651	0.1218	0.0365
